# The inhibitory network of the striatum at the edge of chaos

**DOI:** 10.1186/1471-2202-14-S1-O19

**Published:** 2013-07-08

**Authors:** Adam Ponzi, Jeffery R Wickens

**Affiliations:** 1 Okinawa Institute of Science and Technology (OIST), Okinawa, Japan

## 

The striatum forms the main input structure to the Basal Ganglia (BG), a subcortical structure involved in the selection and reinforcement learning of action sequences. It is 90% composed of medium spiny neurons (MSNs) which inhibit each other through collaterals, receive excitatory projections from cortex and are the only cells projecting outside the striatum. Because of its inhibitory structure the MSN network is often thought to act selectively, transmitting the most active cortical inputs downstream in the BG while suppressing others. However studies show that local MSN network connections are too sparse and weak to perform global selection and their function remains puzzling. Here we suggest that rather than generating a static stimulus dependent activity pattern the MSN network is optimized to generate stimulus dependent dynamical population activity patterns for extended time periods after variations in cortical excitation. Indeed MSNs form cell assemblies whose population firing rates vary coherently on slow behaviourally relevant timescales [1]. Furthermore individual MSNs display diverse response profiles locked to task and reward predicting events [2,3] with phasic activity peaks broadly distributed across the whole spectrum of delays after task events [4-6]. We have previously shown [7,8] that such activity emerges in a model of a spiking MSN network but only at realistic connectivities of ~15% and only when MSN generated inhibitory post-synaptic potentials (IPSPs) are realistically sized. Here we suggest a reason why the MSN network generates such activity. We investigate how network generated population activity interacts with temporally varying cortical driving activity, as would occur in a behavioral task. We find [9] that at unrealistically high connectivity a stable winners-take-all regime is found where network activity separates into fixed stimulus dependent regularly firing and quiescent components. In this regime only a small number of population firing rate components interact with cortical stimulus variations. Around 15% connectivity a transition to a more dynamically active regime occurs where all cells constantly switch between activity and quiescence. In the low connectivity regime MSN population components wander randomly and here too are independent of variations in cortical driving. Only in the striatally realistic transition regime do weak changes in cortical driving interact with many population components so that sequential cell assemblies are reproducibly activated for many hundreds of msecs after stimulus onset and PSTH display strong stimulus and temporal specificity. We show that this activity is maximized at striatally realistic connectivities and IPSP sizes and cortical stimuli are also maximally distinguished at striatally realistic parameter settings. In fact Lyapunov exponent computations show that, quite remarkably, the MSN network sits precisely at a marginally stable point, the edge of chaos. Thus we suggest the local MSN network has optimal characteristics - it is neither too stable to respond in a dynamically complex temporally extended way to cortical variations, nor is it too unstable to respond in a consistent repeatable way. We discuss how these properties may be utilized in temporally delayed reinforcement learning tasks strongly recruiting the striatum.
